# Shaping Circularity in the Food Industry: Strategic Pillars Enabled by Biorefinery Systems

**DOI:** 10.3390/foods15091600

**Published:** 2026-05-06

**Authors:** Maximilian Espuny, Ana Luiza de Oliveira Maia, Camila Fabrício Poltronieri, Cleginaldo Pereira de Carvalho, Otávio José de Oliveira

**Affiliations:** 1 FEG—Faculty of Engineering and Sciences, UNESP—São Paulo State University “Júlio de Mesquita Filho”, Guaratinguetá 12516-410, Brazil; ana.l.maia@unesp.br (A.L.d.O.M.); cleginaldo.carvalho@unesp.br (C.P.d.C.); otaviodeoliveira@uol.com.br (O.J.d.O.); 2 Lorena School of Engineering (EEL), University of São Paulo (USP), Lorena 12602-810, Brazil; camilafp@usp.br

**Keywords:** circular economy, food industry, biorefineries, waste valorization, sustainable food systems, high-value products

## Abstract

Food systems are currently challenged by a difficult balance: they rely heavily on natural resources while simultaneously generating significant volumes of waste, all under increasing pressure to decarbonize operations and close material loops. In this context, this study proposes strategic pillars for circular practices in the food industry, with an emphasis on the transformation of waste and by-products into high value-added resources through bio-based processes supported by biorefineries, in line with the Sustainable Development Goals (SDGs). To underpin this proposal, a PRISMA-guided content analysis of the literature published between 2019 and 2024 (Scopus) identified 30 recurrent CE elements. These elements were systematized into five strategic pillars: valorization of residues and by-products; digitalization of the food supply chain; sustainable education and stakeholder engagement; strategic partnerships for circular business; and regenerative practices based on renewable resources. Together, these pillars point to practical pathways, including the reuse of residues to produce functional ingredients and nutraceuticals, the creation of innovative, sustainable packaging, the generation of renewable energy from biomass, the strengthening of local supply networks, and the use of digital technologies to enhance traceability and transparency. By integrating and organizing fragmented evidence, the proposed framework delivers effective guidance to food industry actors, helping overcome economic and operational barriers to circular practices while supporting collaboration with local partners and research institutions. In doing so, it additionally contributes to advancing key SDGs, particularly SDGs 2, 7, 9, 12, 13, and 17.

## 1. Introduction

The global food production system is moving in the opposite direction of eradicating world hunger, since approximately 931 million tons of food are wasted annually [1,2], while more than 800 million people continue to suffer from food insecurity [3]. This paradox reveals profound structural failures within the food industry: production expands under market forces, yet losses continue across supply chains, with severe environmental effects. The logic of overproduction, driven by industrial models which focus on continuous growth in supply, contributes to waste throughout the chain and intensifies the environmental impacts of overexploiting natural resources—evident in the indiscriminate use of fertilizers and pesticides, deforestation for agriculture expansion, large-scale livestock farming, and long-distance transportation. These dynamics accelerate soil degradation, exacerbate water scarcity, and contribute to biodiversity loss [4,5,6,7].

Despite its key role in human survival, the food sector paradoxically stands among the major contributors to those systemic dysfunctions. The linear economic model—based on extraction, production, consumption, and disposal—has predominated since the Industrial Revolution, but now shows clear signs of exhaustion amid present ecological and social crises [8]. As a result, leading global actors have increasingly called for a rethinking of the concepts that sustain food production chains, demanding structural transformations that go beyond incremental improvements [9]. Within this context, Circular Economy (CE) has appeared as an effective alternative to overcome the weaknesses of linear production and consumption systems, as it is framed as a restorative and regenerative economic logic that seeks to reduce the inflow of virgin materials and decrease waste, emissions, and losses [10]. Aligned with sustainable development principles, CE presupposes integrating environmental, economic, and social aspects to reshape production and consumption models toward more circular, resilient, and fair supply chains [11]. Accordingly, CE is not limited to process efficiency; it is a structural transformation in how value is created, maintained, and distributed over time, reconciling economic activity with environmental protection and social fairness [12].

The interdependence among economic, environmental, and social elements is especially clear in food systems, which encompass agricultural production, industrial processing, distribution operations, trade, consumption, and the management of waste generated across all stages. Each link brings specific sustainability challenges, increasing the complexity of circular transition routes [4,13]. Agriculture plays a central role in this configuration: it employs more than 1 billion people and accounts for approximately 10% of global GDP [3]. Environmentally, it accounts for around 30% of global greenhouse gas emissions and consumes over 70% of global freshwater resources [3,5]. Socially, about 60% of agricultural workers worldwide are employed under informal conditions, without fundamental rights associated with decent work [4]. These signals indicate that the prevailing structure of food production—from agriculture to industry and distribution—remains heavily dependent on natural inputs and production practices oriented toward volume and cost, often externalizing ecological limits and social costs.

Although CE has become increasingly present in institutional discourse and sectoral agendas [3,14], its implementation in food systems is limited. Internationally, circularity has progressively moved from a conceptual aspiration to a political priority, particularly in the European Union. Major initiatives such as the European Green Deal [15], the Circular Economy Action Plan [16], and the Farm to Fork Strategy [17] frame the transition of food systems as a central component of climate-neutrality and sustainable-growth agendas. Instead of treating waste reduction and resource efficiency as isolated objectives, these programs advocate a systemic reshaping of agri-food chains, encouraging the cascading use of bio-based resources, the valorization of biomass, and the incorporation of bioeconomy principles at all stages of production and consumption. In parallel, the Sustainable Development Goals [18] reinforce the need to align food production with broader commitments to conscious consumption (SDG 12), climate action (SDG 13), zero hunger (SDG 2), and partnership-driven transformation (SDG 17). However, while these strategies define an institutional direction, translating political objectives into industrial-scale implementation remains a constant challenge. Barriers frequently cited include the low economic value of waste, high reverse logistics costs, and resistance to incorporating circular practices into traditional production chains [19]. In addition to financial costs, regulatory constraints, a lack of technical capabilities, and the complexity of coordinating stakeholders along supply chains, these factors can also limit the advancement of the CE in the food industry. These challenges highlight that the transition to circular systems involves aspects beyond technology, including institutional and organizational processes, and requires alignment with various internal and external stakeholders [20,21,22,23]. Conquering these obstacles depends on structural transformations involving public policies, business model innovation, and initiatives intended at changing consumption behaviour [24]. Yet, in bio-based production systems, “structural transformation” cannot be confined to strategy and governance alone. Circularity in food systems also depends on operational infrastructure capable of converting heterogeneous biomass streams into multiple value outputs at an industrial scale. In practice, many agro-industrial residues still have substantial biochemical potential—carbohydrates, proteins, lipids, fibres, and phenolic compounds—yet remain underutilized due to restricted processing infrastructure and scale constraints. Conversion routes such as enzymatic hydrolysis, microbial fermentation, anaerobic digestion, thermochemical treatments (e.g., pyrolysis and gasification), and advanced extraction techniques have shown the capacity to transform low-value waste into biofuels, bio-based chemicals, and functional food ingredients. However, the feasibility of growing these routes depends on integrated industrial architectures that coordinate feedstock supply, conversion processes, and market outlets [25,26]. This coordination is especially important in the food industry, because agro-industrial processes continuously produce large volumes of organic waste that share biochemical characteristics similar to those of biomass traditionally processed in biorefineries. Biorefineries are thus seen not as separate or parallel industrial systems but as complementary infrastructures that can link food processing operations with pathways of biomass conversion in order to realize circularity in food-based industrial systems [27,28].

In this context, the concept of biorefineries appears to be a relevant operational tool for boosting CE implementation in the food sector. Similar to oil refineries, biorefineries integrate biochemical, thermochemical, and biological conversion pathways to process diverse biomass streams—including agro-industrial waste, lignocellulosic materials, and food-processing byproducts—into bioenergy, biochemical products, functional ingredients, and biomaterials. By enabling the cascading use of biomass, biorefineries can support the circularity of bio-based value chains and strengthen the integration of renewable energy [26,29,30]. Moreover, unlike linear processing models, biorefinery systems are characterized by a multi-product logic that creates multiple value streams from a single biological resource, improving the economic viability of conversion processes and supporting the consolidation of bio-based value chains when circularity is integrated into system design [31,32].

Alongside infrastructure, circular transition also requires organizational and technological innovations within food chains. Practices such as upcycling—transforming residues into higher added-value products—have been highlighted as important tactics to reduce waste and valorize agro-industrial byproducts [33]. In parallel, digital technologies, including blockchain and the Internet of Things (IoT), have become prominent tools to improve traceability and transparency, reduce resource use, and support reverse logistics by enabling real-time monitoring throughout product life cycles [19,34]. Still, the shift toward circularity demands wider systemic changes, including cultural and institutional transformations and the establishment of economic incentives capable of driving large-scale adoption [35,36].

Recent studies have expanded the debate on CE implementation in food systems, yet important integration gaps continue [37]. For example, systematic evidence on food loss and waste within CE indicates that a limited perspective still prevails, often centred on reuse and recycling rather than comprehensive transformations across production chains [38]. Other research stresses the role of ongoing innovation and organizational adaptability as enabling conditions for circular practices in agri-food companies [39]. Mapping studies also highlight theoretical fragmentation and reinforce the need to integrate CE into environmental, economic, and community dimensions of strategies and practices if it is to contribute efficiently to sustainability outcomes [40,41]. In addition, CE-oriented approaches to sustainability in the food and beverage sector often acknowledge that operationalizing them is still a major challenge [42]. Taken together, these contributions suggest that, in spite of advances in circular practices and biomass conversion knowledge, a fragmentation persists between strategic CE frameworks which articulate what should be done to advance circularity and operational infrastructures that define how it can be done at industrial scale [14,19,40]. Moreover, studies frequently emphasize waste handling procedures, decision-making procedures, or isolated technologies, but less often connect biorefinery-based infrastructures to structured strategic pillars capable of guiding sector-wide circular transformation [24,38,42]. This disconnection weakens the translation of bioindustrial potential into implementable transitional pathways for food production systems.

To address this gap, this study asks: How can the circular transition in food production be fostered through these strategic pillars, and how can biorefinery infrastructure strengthen them at scale? Accordingly, the main objective of this study is to propose strategic pillars for implementing circular practices in food production, while specifying how biorefinery systems can operationally reinforce biomass valorization and bioorganic value chains aligned with the Sustainable Development Goals (SDGs) 2, 7, 9, 12, 13, and 17. To support this objective, the study consolidates and analyzes recent advances in CE strategies in the food industry, especially those related to the valorization of residues and by-products. In addition to this introduction, Section 2 presents the theoretical background supporting the discussion on circularity and the enabling role of biorefineries in food systems. Section 3 describes the research method. Section 4 presents the identification and classification of structuring elements according to SDGs. Section 5 proposes strategic pillars for CE transition in the food industry. Finally, Section 6 presents conclusions, contributions, and directions for future research.

## 2. Theory Background

### 2.1. Circular Economy Foundations in the Food Industry

CE plays a central role in restructuring the food industry, one of the most critical sectors for society and the economy, yet deeply marked by structural contradictions. Although it sustains billions of people worldwide, this sector also generates significant waste, consumes natural resources intensively, and distributes benefits unequally along the production chain. In this context, CE proposes a new production model based on restoring material flows, reducing losses, and environmental regeneration, essential elements for the food industry to take a leading role in the transition to sustainable systems. By incorporating the principles of CE, the food sector is strengthened to overcome the fragmented logic that has historically separated production efficiency and environmental responsibility, promoting models that integrate technological innovation, circularity, and socio-environmental justice [43,44]. For the implementation of CE in the food industry, five fronts stand out: product redesigning and waste recovery [45]; digitization and traceability for flow optimization [46]; integration between operational management and environmental governance [24]; cultural changes aimed at conscious consumption [19]; and the adoption of regulatory instruments and performance metrics that enable large-scale circularity [47].

The coordination between operational management and environmental governance deserves special clarification, since the circular economy is still frequently interpreted as an environmental agenda oriented on waste and emissions. The circular transition of agri-food systems requires adopting environmental criteria into operational planning, supply chain coordination, and performance measurement frameworks, rather than treating sustainability as an external layer of compliance [39,48]. From this perspective, governance mechanisms must align strategic sustainability commitments with day-to-day production decisions and material-flow management. In this way, the circular economy operates as an integrative management logic that synchronizes ecological performance, economic viability and organizational supervision within industrial systems [49,50].

### 2.2. Operational and Governance Dimensions of Circular Transition

Product redesign in the food industry, according to CE, involves altering inputs, processes, and packaging to extend shelf life, reduce waste, and effectively reincorporate residues into production. The use of by-products from fruit and vegetable processing, such as peels, pulp, and seeds, in the production of flours rich in fiber, antioxidants, and bioactive compounds is often used in the preparation of baked goods and processed foods with enhanced nutritional properties [51]. The recycling of food waste involves its technical transformation into new ingredients or higher-value components. Research indicates, for example, the use of vegetable waste rich in soluble and insoluble fiber to create functional foods that have thermal stability and palatable sensory properties [52]. On the other hand, packaging is undergoing reformulation to emphasize recyclable materials and biodegradable structures, meeting requirements for preservation, reduced environmental impact, and traceability after consumption [53].

Digitization and traceability for flow optimization have mainly occurred through the implementation of digital technologies such as the IoT, blockchain, artificial intelligence (AI), sensors, and big data, stimulating the circular transition in the food sector. These technologies enable constant monitoring of processes, traceability of materials, and integration of information throughout the entire supply chain, from initial production to final consumption [53,54]. The IoT has enabled connectivity between devices across the supply chain, enabling remote monitoring of essential variables such as temperature, humidity, and transport time. This real-time data allows preventive loss management and decision-making focused on food preservation and safety. On the other hand, blockchain enables the storage of information in a decentralized, immutable manner, enabling accurate traceability of raw materials and products and simplifying reverse logistics, recalls, and food redistribution procedures [52]. AI has been used to forecast demand, dynamically adapt production, and detect waste patterns. This analytical capacity is enhanced by sensors in packaging, vehicles, or inventory systems that continuously send relevant operational data. Combining this data with big data tools enables consolidating indicators and identifying logistical obstacles, encouraging more effective flows aligned with circular principles [53].

Furthermore, implementing CE in the food sector requires integrating operational management and environmental governance to ensure that circularity principles are incorporated into both daily routines and strategic guidelines. This integration requires adopting more sustainable production systems, standardizing in line with norms such as ISO 14001 [55], and establishing performance indicators to evaluate resource use, waste generation, and energy efficiency [56]. At the organizational level, environmental governance involves structures that align sustainability goals with organizational culture to promote the active participation of leaders and suppliers and ensure consistency between circular commitments and control and transparency mechanisms [54].

Another important aspect is the cultural shift towards conscious consumption, which involves how consumers perceive, select, and assign value to food. Some barriers, such as food neophobia—aversion to new products or those acquired through unfamiliar technologies—directly affect the acceptance of recycled ingredients, even when they are linked to environmental benefits [57]. This resistance is intensified by perceptions of danger, misinformation, and lack of ecological knowledge, particularly in situations where the principles of circularity are poorly understood [54]. In this context, access to information, particularly digital sources, is essential for shaping consumer awareness and attitudes towards responsible food practices. When consumers have access to information, they can significantly expand their environmental knowledge and become more likely to adopt innovative or circular products; however, this does not necessarily imply consistent behavioural change, as decision-making remains influenced by trust, perceived risks, and contextual factors [58].

Finally, adopting standards and metrics to guide and evaluate circularity across the production chain is essential to consolidating CE in the food industry. Standards such as ISO 14040 [59] and 14044 [60], which are associated with Life Cycle Assessment (LCA), have been widely used to assess the environmental impacts of food products. To simultaneously parameterize the environmental, material, and economic dimensions, the Material Flow Analysis (MFA) and Life Cycle Cost Analysis (LCCA) tools are also being used. While MFA enables assessing resource efficiency and material circulation within food systems, LCCA helps evaluate the economic viability of circular strategies over time [61,62,63]. In addition to the tools, it is worth highlighting the Product Environmental Footprint (PAP), which, in turn, enables comparisons across food categories using integrated performance indicators, such as resource use, energy intensity, and waste reintegration [64]. In general, these tools support certifications, ESG goals, and strategic decisions by quantifying key aspects of circularity, such as material reuse efficiency and the presence of by-products in food formulations.

The combination of CE principles and the specificities of the food industry provides a solid theoretical basis for reconfiguring production systems toward more regenerative and integrated models. In this process, approaches centred on reorganizing material flows become increasingly relevant, especially when aligned with institutional and cultural transformations that reinforce governance mechanisms, stimulate social engagement, and broaden the distribution of responsibilities across the value chain [65]. These transformations require transdisciplinary strategies that articulate environmental sustainability, economic viability, and social equity in concrete, verifiable practices [64]. At the same time, implementing circularity in the food sector requires overcoming structural obstacles, strengthening regulatory frameworks, and promoting a collaborative culture among the actors in the agri-food system [48].

While operational and governance mechanisms structure the organizational dimension of circular transition, the materialization of circularity in bio-based sectors ultimately depends on the availability of integrated technological infrastructures capable of valorizing heterogeneous biomass streams at scale.

### 2.3. Biorefinery Systems and Biomass Conversion as Structural Enablers of Circularity

Biorefinery systems are pivotal as technological enablers for the application of EC principles in bio-based industries. Analogous to oil refineries, biorefineries integrate a range of biochemical, thermochemical, and biological conversion pathways to convert heterogeneous biomass streams into energy, bio-based chemicals, materials, and functional compounds [25,26,29]. Contemporary biorefineries are characterized in the IEA Bioenergy Task 42 (2022) as being constructed on the principle of cascading utilization of biomass and the valorization of numerous products to boost resource utilization and reduce waste streams [30]. From a biochemical engineering perspective, the efficiency of biomass conversion in biorefinery systems depends strongly on how key process parameters are controlled, particularly those governing reaction kinetics and mass transfer phenomena [27].

In biochemical conversion routes, enzymatic hydrolysis depolymerizes lignocellulosic structures into fermentable sugars under relatively mild processing conditions. At this stage, substrate–enzyme interactions, enzyme loading, and operating conditions such as temperature and pH directly influence conversion rates and sugar yields, typically described through kinetic models such as Michaelis–Menten behaviour [66]. Although pretreatment is often required to degrade lignin-carbohydrate complexes, the yield and economic sustainability of the processes have been well advanced through optimization of enzyme activity and, to some extent, industrial biotechnology [26]. Such fermentable substrates will later, via microbial fermentation, be transformed into bioethanol, lactic acid, succinic acid and a class of basic chemicals that make up bio-based chemical production chains [25]. During microbial fermentation, maintaining a balance between substrate availability, metabolic pathway efficiency, and process stability is critical, as these factors jointly determine conversion yields, productivity, and product selectivity [67]. In industrial settings, these parameters directly influence scalability and economic viability [68].

Pyrolysis and gasification, via different thermochemical routes, can convert lignocellulosic residues and heterogeneous food waste into bio-oil, synthesis gas, and biochar at controlled temperature conditions, thereby increasing their flexibility as feedstocks. Fast pyrolysis processes, especially, have proven technical viability for generating liquid biofuel precursors, making it feasible to valorize fractions of biomass less suited to biochemical processing. These pathways promote systemic resilience by diversifying product portfolios and lessening reliance on a single-product processing pathway [29].

In parallel, extraction technologies—including solvent extraction and emerging techniques such as supercritical CO_2_ extraction—enable the recovery of high-value-added bioactive compounds, phenolics, fibres, lipids, and proteins from fruit, vegetable, cereal, and dairy waste. The efficiency of these processes depends strongly on operational parameters such as solvent selection, temperature, pressure, and extraction time, which directly influence both recovery yields and product quality [69]. Advanced techniques such as supercritical fluid extraction have gained increasing attention due to their ability to enhance selectivity while reducing environmental impacts [70]. These processes are particularly relevant in the food sector, where functional ingredients and nutraceutical compounds represent high-value products capable of improving economic viability [30]. In this context, nutraceuticals are usually referred to as food-centred products or components that offer health benefits beyond basic nutrition, which can be attributed to their bioactive constituents (such as polyphenols, fibres, peptides, and antioxidants) in food systems. Nutraceuticals, unlike pharmaceuticals, are generally derived from natural food matrices and are also added to functional foods, supplements, or other products at the nutrition/health interface. Their increased industrial acceptance has raised interest in the value of agro-industrial by-products as an alternative, sustainable source of nutraceuticals, particularly when supported by biorefinery-based extraction and purification techniques [71].

**Figure 1:**
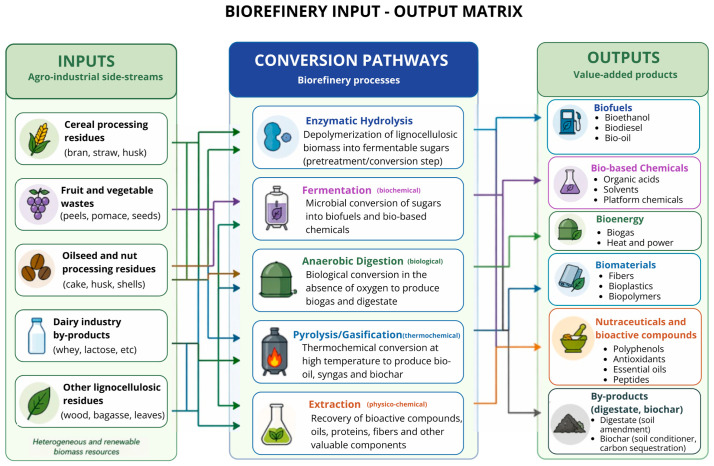
Conceptual biorefinery input–output matrix linking agro-industrial side-streams, conversion pathways, and value-added products within circular bioeconomy systems. Source: Elaborated by the authors based on literature synthesis [29,30,68,69,70].

To further illustrate the systemic organization of biorefinery systems, Figure 1 presents a conceptual input–output matrix linking biomass feedstocks, conversion pathways, and value-added products. This representation highlights the cascading utilization of biomass and the integration of multiple technological routes within circular bioeconomy systems.

Figure 1 presents an integrated view of biomass inputs, conversion pathways, and value-added products in biorefinery systems, highlighting the cascade logic and underlying principles of circular bioeconomy. From an evaluation perspective, integrating Life Cycle Assessment (LCA) and Techno-Economic Analysis (TEA) enables a systematic comparison of the main biorefinery pathways by jointly considering environmental and economic performance. In particular, indicators such as energy intensity, greenhouse gas emissions, and economic feasibility are critical for assessing the sustainability of biomass valorization strategies, as widely discussed in the biorefinery literature [29,72].

As summarized in Table 1, different conversion pathways exhibit distinct performance profiles, reflecting inherent trade-offs between environmental impact, resource efficiency, and economic viability. This comparative synthesis highlights that no single pathway is universally optimal, reinforcing the need for context-specific decision-making in the design of circular biorefinery systems.

The assimilation of these innovative technological platforms into multi-product biorefinery systems constitutes a structural shift from linear waste management approaches. Integrated biorefineries not only design for efficient use of such end-of-line materials such as biomass, but also design industrial systems to produce multiple value streams from a single biomass rather than treating waste as a finished product. This cascade logic leads to better resource utilization and facilitates bio-based value chains that support circularity at the design stage of the process [29].

Even though we are making technological progress, many structural obstacles continue. Variability of raw material and seasonal availability constrain the homogeneous application of technologies; changing from the laboratory or factory scale to pilot and commercial scales necessitates considerable capital costs and proprietary engineering expertise; and economic performance depends on variations in fossil fuel prices and political incentives [26,30]. Thus, the transition to bioindustrial circularity in the food industry depends on technical feasibility, the systemic integration of conversion platforms, coordinated industrial infrastructure, and governance mechanisms that support bio-based value chains with multiple products [72].

Alongside earlier technological and process improvements, recent progress in Industry 4.0 and AI now plays a major role in optimizing biorefinery operations. Using digital tools such as sensors and IoT systems, operators can continuously collect data at every stage of biomass conversion [77,78]. These technologies help automate processes and improve control, making it easier to adjust key factors such as temperature, pressure, and reaction time. AI methods, such as machine learning and predictive modelling, are now often used to boost fermentation efficiency, predict yields more accurately, and lower process variability [79,80]. Digital twins and data-driven decision systems also enable the simulation and optimization of biorefinery setups, helping to use resources more efficiently and better connect different processes [77,81]. These advances improve both environmental outcomes and economic outcomes, underscoring the importance of Industry 4.0 in advancing circular and bio-based industries [68,82].

## 3. Research Method

This research uses a qualitative, exploratory, and descriptive method grounded in content analysis. This methodological choice is justified by the complexity and multidimensionality of the topic under study—the CE applied to the food sector—and by the need to critically analyze a vast body of textual evidence to identify organizational patterns and strategies aligned with the Sustainable Development Goals (SDGs). According to [83], content analysis is a set of methods that enable valid and reproducible inferences from textual information, assisting in the formation of thematic categories and the detection of meanings implied in the messages examined.

Content analysis, in addition to being widely used in studies on sustainability, digital transformation, and production systems, has established itself as an efficient tool for developing interpretive protocols for organizational practices focused on circularity, particularly when linked to references such as the Agenda 2030. Recent research illustrates the soundness of this strategy in scenarios involving structural changes in production chains, highlighting its usefulness in connecting business practices with global sustainable development guidelines [84,85,86].

The assessment was conducted using a hybrid approach, combining a deductive methodology based on the 17 Sustainable Development Goals suggested by the UN [87] with an inductive method focused on detecting emerging elements directly from the document collection, as indicated by Elo et al. (2014) [88]. This approach enabled the integration of established sustainability categories with discoveries grounded in the realities of the food industry, providing greater analytical depth and methodological precision to the study.

**Figure 2:**
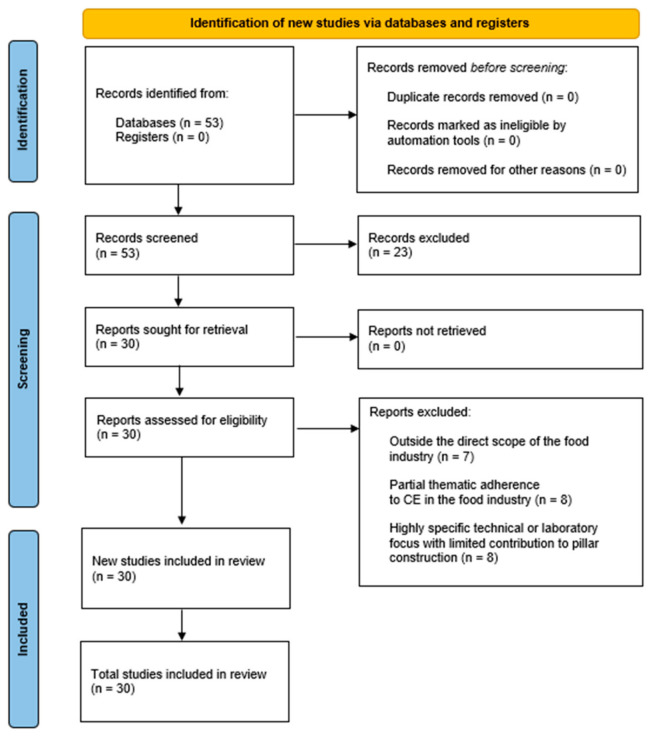
PRISMA 2020 flow diagram of the study selection process. The diagram illustrates the identification, screening, eligibility assessment, and inclusion of studies, including explicit exclusion criteria aligned with the scope of CE in the food industry.

The corpus selection phase was carried out in accordance with the PRISMA (Preferred Reporting Items for Systematic Reviews and Meta-Analyses) protocol [89], which is known for ensuring transparency, traceability, and replicability in systematic reviews, including qualitative research (Figure 2). The research was conducted on the Scopus platform, using Boolean operators in the document title and keyword fields. The terms used were: “circular economy” OR “circularity” OR “circular design” AND “food production” OR “food industry.” The inclusion criteria were defined as follows: (1) articles and review papers; (2) published between 2019 and 2024; (3) written in English; and (4) indexed in the Scopus database. Therefore, the string used in Scopus in the search was: “(TITLE (“circular econom*” or “circularity” or “circular design”) OR AUTHKEY (“circular econom*” or “circularity” or “circular design”) AND TITLE (“food production” OR “food industry”) OR AUTHKEY (“food production” OR “food industry”)) AND PUBYEAR > 2015 AND PUBYEAR > 2018 AND PUBYEAR < 2024 AND PUBYEAR > 2018 AND PUBYEAR < 2024 AND (LIMIT-TO (DOCTYPE,”ar”) OR LIMIT-TO (DOCTYPE,”re”)) AND (LIMIT-TO (LANGUAGE,”English”))”. The exclusion criteria included duplicate records, documents not directly related to the food industry, and studies not available in full text.

**Figure 3:**
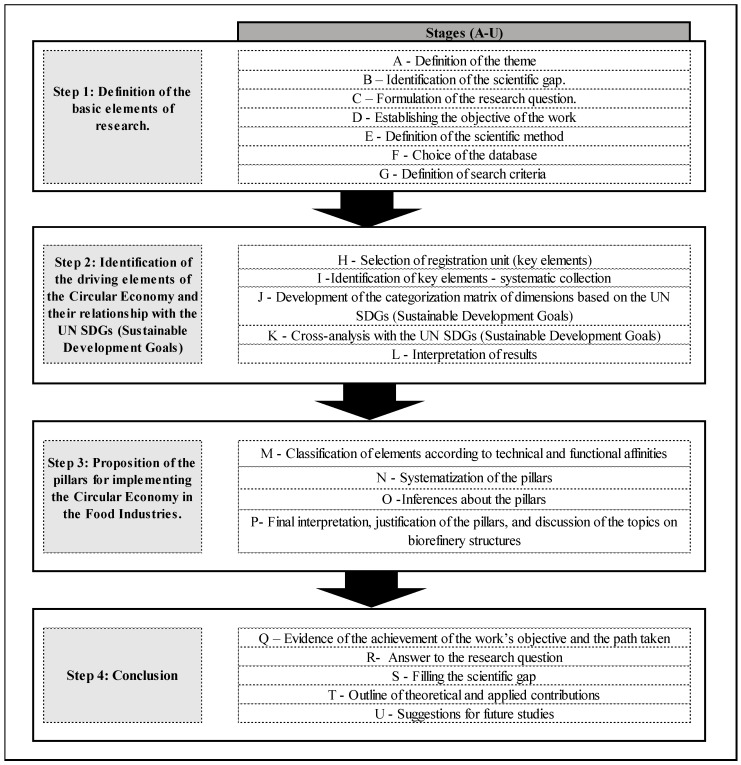
Methodological Workflow. Design of the methodological flow developed in this work. Phase 1—Definition of the basic research elements; Phase 2—Identification of the structuring elements of the CE and their relationship with the SDGs. Phase 3—Proposition of the pillars of the CE in the Food industries; Phase 4—Elaboration of the Conclusion.

The initial search yielded 53 documents, of which 30 were selected based on two additional criteria: (1) minimum theoretical and empirical adherence to the research topic—which involves the connection between circularity practices and the food industry—and (2) the number of citations, used as an indicator of the scientific impact and importance of the works. We consider the criterion of “minimum theoretical and empirical adherence” to be the extent to which the selected studies explicitly address circular economic practices in the food industry, either through conceptual discussions, empirical analyses, or case-based applications. The connection between circular economic practices and the food sector was operationalized by identifying studies that directly examine topics such as resource recovery, waste valorization, circular production models, and sustainability strategies applied to agri-food systems. The number of citations was used as a complementary indicator of scientific relevance, supporting the selection of studies with recognized academic impact, without being applied as a strict exclusion threshold. The selected articles formed the final sample for analysis, from which the structuring elements of the circular economy in the food sector were extracted. The selected articles formed the final sample for analysis, from which the structuring elements of the CE in the food sector were extracted. These elements were then organized into preliminary thematic groupings and linked to the Sustainable Development Goals (SDGs). The association between the identified structuring elements and the SDGs was carried out as a complementary analytical step, aiming to contextualize the relevance of the findings for sustainability. Given the exploratory nature of the study, each element was linked to the SDG with which it showed the greatest conceptual alignment to preserve clarity and avoid overly overlapping interpretations. This procedure was performed through iterative interpretation and cross-validation among the authors, ensuring internal consistency and theoretical alignment. The link between the structuring elements and the SDGs is therefore interpreted as a conceptual alignment rather than a direct measure of impact. In this sense, the potential contributions of CE practices to specific SDGs are considered context-dependent and may involve trade-offs, depending on technological, organizational, and regulatory conditions. A visual illustration of the method is presented in Figure 3.

### 3.1. Step 1—Defining the Basic Elements of the Research

The initial stage involved defining the principles that guided the research, including delimiting the topic and selecting the method. At the beginning (Stage A), the study evaluated CE practices in the food industry, with an emphasis on identifying the structuring elements aligned with the Sustainable Development Goals (SDGs). Subsequently (Stage B), a scientific gap was identified through a critical analysis of the literature presented in the Introduction, which revealed persistent fragmentation in studies on CE in the food sector. In particular, previous research tends to address circular practices, waste management, or technological solutions in isolation, with limited integration between strategic frameworks and operational infrastructures, especially in relation to biorefinery systems and their alignment with the SDGs. Subsequently, the research question was formulated (Stage C), and the study’s objective was established (Stage D).

The decision to use content analysis as the research methodology was made in Stage E, based on its effectiveness in qualitatively processing large amounts of textual data and identifying relevant thematic categories [83,88]. In Stage F, the Scopus database was selected as the data source for its broad coverage, accuracy, and global indexing, particularly for high-impact publications in sustainability and innovation.

Concluding this phase, in Stage G, the research criteria that guided the scientific scope were established: the use of descriptors in the title and keyword fields (“circular economy,” “food industry,” among others), with a time interval from 2019 to 2023, in English, and documents categorized as scientific articles or reviews.

### 3.2. Step 2—Identification of the Structuring Elements of CE and Their Relationship with the SDGs

The second stage began with Stage H, which defined the unit of record: the structuring elements of CE in the food industry. In this study, the structuring elements represent actions, strategies, or technologies related to circularity in the food sector. Next, Stage I consisted of reading the documents in their entirety and systematically collecting these components. The evaluation sought to identify passages that could symbolize good practices, innovations, obstacles overcome, or organizational guidelines for material reuse, waste reduction, by-product valorization, digitization, traceability, sustainable packaging, and bioenergy, among other characteristics. The collected excerpts were organized into an analytical table, preserving the original context and references.

Based on this information, we moved on to Stage J, where the elements identified in the previous steps were organized in a categorization matrix guided by the SDGs. Each structuring element was associated with the 2030 Agenda goal(s) that best reflected its nature and intended function. The SDGs identified in this study represent those most frequently associated with circular economy practices in the analyzed literature and should not be interpreted as an exhaustive set of all possible CE–SDG relationships. Rather, they reflect an empirical mapping derived from the recurrence and thematic alignment of the structuring elements identified through content analysis. For example, sustainable agricultural practices and the use of waste as a protein source were associated with SDG 2 (Zero Hunger and Sustainable Agriculture); actions aimed at valorizing waste for bioenergy were linked to SDG 7 (Affordable and Clean Energy); and actions focused on reducing waste and reusing food were associated with SDG 12 (Responsible Consumption and Production), among others.

In Stage K, the results of this classification were arranged in a double-entry table, improving the interface for viewing the frequency of elements in each document and their relationship with the Sustainable Development Goals. This mapping enabled us to determine which 2030 Agenda goals are most prevalent in the literature examined and to identify which topics exhibit the highest incidence of circular practices in the food sector. At the end of this stage, in Stage L, the consolidated data underwent a first interpretive inference, aimed at understanding the meaning and function of each component in the circular logic and its relationship with global sustainability commitments.

### 3.3. Step 3—Systematization and Proposal of Pillars for Implementing CE in the Food Industry

After identifying and classifying the structuring elements, the third stage involved arranging the data into thematic axes based on conceptual affinity, thereby structuring pillars that guide the implementation of CE in the food industries. This stage was guided by an inductive qualitative study, based on both the frequency and analytical density of the elements already coded.

In Stage M, the structuring elements were reorganized based on their thematic and functional affinities, considering the domains in which they operate within the circular logic—such as production processes, supporting technologies, business models, reuse strategies, and institutional relationships. This classification prioritized internal coherence and conceptual consistency across the groupings, enabling a more robust, synthetic analytical arrangement of the findings.

This process supported the systematization of the pillars in Stage N, consolidating aggregated thematic groupings into higher-order analytical dimensions that represent core directions for advancing circularity in the food sector. At this stage, each pillar also had its corresponding action axes defined, representing the subdimensions through which the pillar unfolds conceptually and operationally. Each pillar was structured into a set of action axes and associated structuring elements (#), articulated through complementary objectives and functions.

Stage O comprised the formulation of the first analytical inferences about the coherence, internal density, and consistency of each identified pillar. The degree of thematic coverage, the complementarity among the grouped elements, and the operational clarity of the structured sets were evaluated to avoid redundancies and ensure more precise conceptual delimitations.

In Stage P, the analyses resulting from pillar identification were deepened by comparing them with the specialized literature, enabling validation and refinement of the proposed thematic groupings. This stage strengthened the theoretical basis of the pillars, aligning the empirical findings with perspectives already consolidated in EC and highlighting specific contributions characteristic of the food sector. The final structuring of these pillars served as the basis for analyzing the results and formulating recommendations applicable to the industrial reality. Furthermore, a discussion was conducted regarding how each of these pillars relates to biorefinery structures.

### 3.4. Step 4: Conclusions

The fourth and final phase aimed to consolidate the results obtained in the previous stages, to indicate how the central research question was addressed, to demonstrate alignment with the previously established objectives, and to discuss the study’s theoretical and practical contributions. In Stage Q, the consistency between the goals outlined at the beginning of the investigation and the analytical paths taken was verified.

Stage R was dedicated to explaining how the research’s guiding question was addressed throughout the process. The stages of identification, categorization, and systematization allowed the empirical data to be articulated analytically, enabling a structured response to the problem defined at the beginning of the investigation. Stage S sought to fill the scientific gap identified in the initial phase. Next, in Stage T, the theoretical and practical contributions of the study were highlighted, and finally, Stage U proposed directions for future studies.

**Figure 4:**
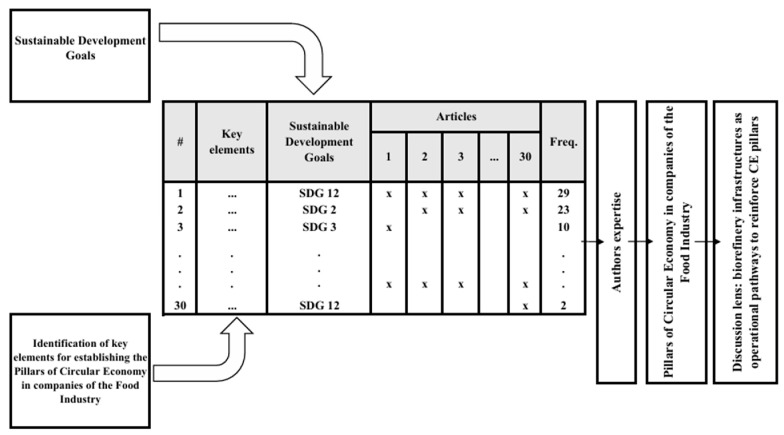
Matrix of Structuring Elements, SDG Alignment, and Article Occurrence. Design of the analytical matrix used to classify the structuring elements identified in the literature. Each element is associated with the Sustainable Development Goal(s) to which it relates and mapped across the 30 analyzed articles, indicating its presence and frequency. This matrix organized theoretical evidence and served as the basis for systematizing the pillars proposed in this study.

The consolidated analytical structure resulting from these stages is summarized visually in Figure 4, which presents the distribution of structuring elements, their alignment with the SDGs, and their recurrence across the analyzed studies.

## 4. Identification and Classification of Structuring Elements According to the SDGs

The adoption of CE practices in the food industry helps prevent environmental damage by reducing waste and reusing organic waste, and by minimizing the release of greenhouse gases and pollutants into the soil, air, and water. In addition, these practices can help reduce hunger by transforming food waste into new, nutritious products, promoting food security, and benefiting vulnerable communities [21].

To drive sustainability forward, the UN proposed Agenda 2030, which, through its 17 Sustainable Development Goals (SDGs), encourages and supports responsible actions across production and consumption, as well as the reuse of waste generated by companies and individuals [87]. These SDGs also support actions to advance the CE in the food industry, promote the reuse of organic waste, develop technological systems for by-product reuse, and form partnerships among diverse actors in society.

These initiatives help reduce the environmental and social impacts of food waste, directly contributing to achieving the goals of Agenda 2030 [87,90]. To achieve the SDGs, there must be integration and cooperation between all sectors of society. The government must establish public policies, laws, and regulations that encourage initiatives to improve the quality of life, raise environmental awareness, and address climate change. The population must be actively involved in formulating public policies and adopting sustainable practices that mitigate environmental impacts.

The 2030 Agenda was adopted in September 2015 by all 193 UN member states to promote a sustainable future for the next generation. Thus, countries, states, and municipalities are free to implement their sustainable development goals, targets, and principles in line with their needs and contexts [87].

Given this flexibility, national, regional, and local governments should support and encourage companies to develop and strengthen CE practices in the food industry, contributing to the UN 2030 Agenda, particularly in achieving SDGs 2, 3, 7, 8, 9, 11, 12, 13, 15, and 17. In this context, the structuring elements identified in the CE framework were individually associated with their corresponding SDGs. Table 2 presents these associations, showing the relationship between each SDG and the specific elements that contribute to its fulfillment. The information that allows the traceability of each element, identified in each article individually, is presented in Table A2.

For SDG 2, which aims to end hunger and promote sustainable agriculture, three elements were identified: encouraging the use of agricultural by-products and waste to generate value in the production chain (E#2); fostering farming practices that reduce the use of chemicals and promote biodiversity (E#13); and using insects to process food and agricultural waste, transforming it into sources of protein (E#19). These elements highlight the importance of optimizing biomass use and generating bioenergy from agrarian waste, reducing the environmental impact of food production, and contributing to a more efficient and sustainable food chain [91].

In addition, encouraging sustainable agricultural practices improves soil resilience and biodiversity, positively impacting the health of workers and consumers by reducing dependence on agrichemicals [92]. Finally, the use of insects to convert waste into protein sources promotes more circular and sustainable food production, contributing to waste reduction and improved efficiency in the food chain [91].

In relation to SDG 3, which aims to ensure healthy lives and promote well-being for all, the two elements identified are: developing educational campaigns that explain the environmental and health benefits of reusing food by-products in the production of new functional foods (E#15); and providing information on how innovative food technologies are used to create food from by-products (E#21). These actions seek to increase consumer acceptance of new products developed from food by-products. The reuse of by-products in the food sector, such as orange juice by-products for flour production, can be incorporated into functional foods, such as cookies and pasta, thereby reducing waste and increasing the nutritional value of these foods [51].

In the context of SDG 7, which aims to ensure access to affordable, reliable, sustainable, and modern energy for all, the three elements identified are: investing in technologies that enable the recycling of agricultural waste (E#4), establishing partnerships with local biorefineries that use anaerobic digestion to convert food waste into renewable energy (E#11), and developing bioreactor technologies that use waste to feed microalgae (E#17).

Agricultural waste can be recycled through processes such as automated composting and pyrolysis, which convert biomass into biofuels, thereby reducing dependence on fossil fuels and supporting the sustainability of the agricultural sector [92]. In addition, partnerships with local biorefineries to convert waste into renewable energy through anaerobic digestion can reduce greenhouse gas emissions and increase energy efficiency [93]. Finally, the development of bioreactors that use waste to feed microalgae enables the lipids and carbohydrates produced by these microalgae to be used for both food production and bioenergy generation [92].

In the context of SDG 8, which aims to promote sustained, inclusive, and sustainable economic growth, as well as full and productive employment, the identified element is to implement technological solutions to monitor the consumption of critical resources (E#7). These solutions promote efficiency and sustainability in the production chain and encourage the adoption of circular business models that integrate Corporate Social Responsibility (CSR) practices, focusing on the reuse and recycling of materials for the well-being of workers and the community. In addition, implementing monitoring in manufacturing processes can optimize energy and water consumption, thereby reducing the environmental impact of production [94]. These systems can also integrate industrial automation processes, enabling greater tracking and control of resources throughout the supply chain [95].

As for SDG 9, which aims to promote innovation and resilient infrastructure, the five elements identified focus on the adoption of sustainable technologies and practices in food production, covering the use of Industry 4.0 technologies to optimize the management of resources and by-products (E#9); investment in research and development of sustainable production models (E#10); the digitization of production processes and the food supply chain using technologies such as blockchain, IoT, and augmented reality (E#12); the adoption of analytical tools to improve transparency and efficiency in waste and by-product management (E#24); and the implementation of industrial-level waste recovery processes (E#29). In relation to the optimization of resource and by-product management, the adoption of tools such as IoT and big data has enabled real-time monitoring of material flows, contributing to more innovative and more sustainable resource management [21]. Investment in research to develop sustainable production models has been essential for addressing demand uncertainties and improving resource management in a CE. The use of statistical models has contributed to promoting the efficiency of material flows, resulting in more resilient and sustainable production [91]. The digitization of production processes and the adoption of technologies such as blockchain and IoT have ensured traceability and food safety, allowing consumers to access reliable information about product origins [21]. About transparency and efficiency, the use of software for waste and by-product management has proven effective in identifying hidden costs and reducing material and energy waste [91]. The implementation of industrial-level waste recovery processes, such as extracting fibers and antioxidants from byproducts, has been a key strategy for transforming waste into new products. This adds economic and nutritional value to by-products, reinforcing the CE cycle [21].

In the context of SDG 11, which seeks to promote more sustainable cities and communities, the identified element prioritizes reterritorializing production, favoring proximity to local suppliers (E#27). This aims to reduce transportation costs, decrease the supply chain’s carbon footprint, support local economies, and increase the supply chain’s resilience. By minimizing the distances between production and consumption points, this practice promotes both environmental sustainability and the economic strengthening of local communities, ensuring more balanced and sustainable development [96].

As for SDG 12, which aims to ensure sustainable production and consumption patterns, the ten elements identified focus on CE practices in the food supply chain, covering waste reduction and reuse of waste in new production cycles (E#1, E#5, E#8, and E#22); awareness and education of consumers and supply chain members (E#3); use of biodegradable and recyclable packaging to minimize environmental impact (E#6, E#16); adoption of clean technologies and green solvents (E#20); redefining business success through degrowth (E#26); and use of social media to promote engagement around the CE (E#30). In relation to waste reduction, the implementation of systems to reuse food waste drives the creation of new products, generating value from by-products [21]. Awareness among consumers and supply chain members, in turn, promotes circular practices by guiding the importance of conscious consumption and recycling [57]. In addition, the use of biodegradable and compostable packaging, such as bioplastics and polylactic acid (PLA), reduces plastic waste in the food supply chain and meets consumer demands for environmentally responsible products [97]. Investing in technologies that use green solvents to produce natural pigments from food waste replaces traditional practices, promoting a circular, sustainable economy in the food sector. The principle of degrowth redefines business success, prioritizing sustainability over financial growth. This implies limiting the use of natural resources, focusing on the efficiency of production processes, reducing environmental impacts, and promoting a more balanced business model [98]. Finally, using social media as engagement tools has become an essential practice for expanding dialogue around the CE, promoting stakeholder education, and fostering collaboration and awareness of sustainable practices in the agri-food sector [97].

In relation to SDG 13, which seeks to combat climate change and its impacts, two elements were identified: the cultivation of algae in aquatic environments to reduce the need for arable land and promote carbon sequestration (E#14); and the establishment of partnerships with local suppliers to minimize emissions associated with product transportation (E#23). Algae cultivation contributes to bioenergy generation, optimizes wastewater use, and promotes the CE in the food sector [56]. The creation of local partnerships strengthens the supply chain, reduces the carbon footprint, and lowers logistics costs, thereby making the sector more efficient and sustainable [99].

In the context of SDG 15, which seeks to protect, restore, and promote the sustainable use of terrestrial ecosystems, the identified elements prioritize the collection and valorization of inedible by-products and the use of fruit-processing waste for bioenergy. Element #25 proposes establishing mechanisms to collect by-products, such as feathers, blood, and slaughterhouse waste, which are traditionally discarded but can be reused in non-food sectors, transforming what would be waste into sources of revenue [21]. Element #28 encourages the use of fruit processing by-products, such as peels, for the production of biochar and bioenergy, closing the production cycle and contributing to a more CE in the food sector [51].

In the context of SDG 17, which aims to strengthen partnerships for sustainable development, element #18 prioritizes forming alliances among food companies, universities, and R&D centers. The goal is to share knowledge, technologies, and best practices on the use of by-products. This enables the public and private sectors to collaborate in seeking sustainable solutions for the food industry, fostering innovation and improving the efficiency of by-product use. These partnerships can accelerate the development of green technologies and circular practices, transforming what would otherwise be wasted into valuable resources for food production [91].

## 5. Proposal of Pillars for the Circular Economy Transition in the Food Industry and Discussions on Biorefinery Structures

The structuring elements identified in Section 4 were systematized and used as the analytical foundation for proposing five strategic pillars of CE in the food industry. While this study focuses on the food industry, it is important to acknowledge that several circular practices described here interact with upstream agricultural processes, reflecting the interconnected nature of agri-food systems. Within the logic of content analysis applied to theoretical material, organizing dispersed conceptual findings into broader analytical structures is essential to ensure coherence and strengthen the explanatory capacity of the synthesis. Accordingly, the structuring elements extracted from the literature were examined for thematic and functional affinities, enabling their consolidation into higher-order analytical groupings—here defined as strategic pillars. This procedure aligns with methodological perspectives that emphasize the importance of aggregating first-order theoretical elements into broader dimensions to enhance clarity, rigour, and conceptual organization [100,101].

Once the pillars were defined, the analysis advanced to a second level of refinement. For each pillar, the structuring elements derived from the literature were reorganized into action axes, which represent subdimensions that specify how the pillar unfolds conceptually. These axes integrate complementary orientations and thematic nuances identified in the reviewed studies, reinforcing the internal consistency and interpretative depth of each pillar.

The resulting strategic pillars were: valorization of waste and by-products; digitization of the food chain; sustainable education and stakeholder engagement; strategic partnerships for circular businesses; and regenerative practices and renewable resources. Each pillar comprises a set of action axes and structuring elements (#), articulated through complementary objectives that collectively support the transition toward more circular and sustainable production models.

**Figure 5:**
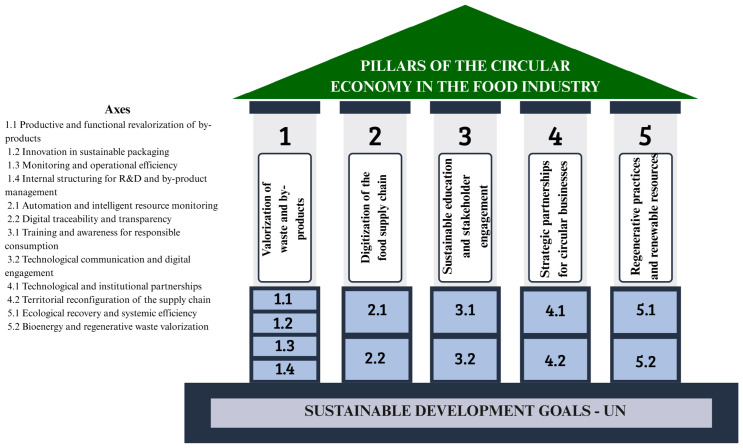
Pillars of the CE in the Food Industry. Visual synthesis of the analytical structure resulting from the clustering of structuring elements, showing the five proposed pillars, their corresponding action axes, and their links to the Sustainable Development Goals (SDGs).

In the following sections, the pillars are presented based on the conceptual evidence highlighted in the analyzed studies, forming a practical instructional set for food industries interested in incorporating circularity principles into their operations. Based on the organization of the structuring elements shown in Table 1 and their thematic affinities, the pillars of CE in the food industry were established as illustrated in Figure 5.

### 5.1. Pillar 1—Valorization of Waste and By-Products

Valorizing waste and by-products is one of the most consistent strategies for operationalizing CE in the food industry. This pillar encompasses organizational practices aimed at reusing materials throughout the production chain, through the reinsertion of waste as productive inputs, the replacement of conventional materials, the setting of environmental goals, and the structuring of internal innovation mechanisms. The structuring elements of this pillar have been organized into four areas of action. In the selected articles, there is significant evidence highlighting the important role of waste valorization as a sine qua non condition for the circular transition, especially regarding resource efficiency and value generation. This evidence is emphasized by valorizing biomass and recovering resources biologically, which constitutes key strategies in circular food systems [52,102]. However, it is stressed that differences in the level of technological sophistication and scalability of solutions may vary from laboratory extraction processes to bio-based systems on an industrial scale [47,103].

The area of productive and functional revaluation of by-products covers practices for transforming food waste into functional ingredients, biomaterials, and industrial inputs (#E2, #E19, #E20, #E25, #E29). The elements analyzed demonstrate, for example, the formulation of ingredients from fibers, legume proteins, and antioxidant compounds extracted from plant waste. Also discussed are processes for converting waste using insects as biotechnological vectors, and technologies that use green solvents to extract natural pigments, reducing dependence on synthetic inputs and adding value to previously discarded waste [47,52,104].

The focus on innovation in sustainable packaging includes the use of biodegradable, recyclable, and compostable materials in the food supply chain (#E6, #E16). It has been found that replacing conventional materials with bioplastics, such as polylactic acid (PLA), reduces plastic waste, promotes market differentiation, and meets the growing demand for environmentally responsible products [105]. In contrast to value-added technologies, packaging innovations tend to be more directly linked to market dynamics and consumer perceptions, playing a complementary role in the circular transition [53,105].

The monitoring and operational efficiency axis includes setting environmental goals, using performance indicators, and adopting tools for waste and by-product control (#E8, #E24). The materials analyzed converge on the application of sensors and digital management systems based on KPIs, aimed at the traceability of by-products and operational transparency. These mechanisms reinforce organizations’ responsibility and the integration of sustainability into decision-making [53,94]. When these two elements are combined, they can boost the effectiveness and scalability of value-added strategies, highlighting the importance of monitoring and performance measurement tools in circular economy systems [21].

The internal structuring axis for R&D and by-product management encompasses actions to organize committees, internal programs, and operational routines to identify and value waste (#E1, #E22). There have been cases in which waste, such as peels, seeds, and inedible fractions, has been systematically analyzed to develop new products. In addition, initiatives include the collection and transformation of inedible waste—such as feathers, blood, and slaughter waste—into inputs for non-food applications, thereby converting environmental liabilities into alternative revenue streams [52,106]. These elements are recurrent in the literature, reinforcing their relevance in the analysis of this pillar [51,107].

Beyond specific technologies, the valorization of waste and by-products in the food industry constitutes a strategic change in how residual biomass is perceived and managed. Instead of being treated as inevitable losses or disposal problems, by-products are increasingly gaining space as secondary resources, as they can generate new economic, environmental, and social value. This perspective promotes reconfiguring food production systems towards integrated value chains, in which waste is systematically reintegrated into production cycles. Therefore, the transition from isolated, dispersed waste-handling methods to systemic valorization pathways requires industrial solutions that can operate at scale and accommodate the inherent heterogeneity of food-derived biomass flows [108,109,110].

From a bioindustrial perspective, the valorization of waste and by-products becomes feasible when supported by integrated biorefinery systems that handle heterogeneous biomass streams and convert them into a range of high-value products [111]. In this context, biochemical biorefineries play a central role by enabling the fractionation of lignocellulosic and protein-rich residues through a sequence of unit operations, including pretreatment, enzymatic hydrolysis, fermentation, and downstream purification. These processes allow the recovery of functional ingredients, bioactive compounds, and bio-derived intermediates that would otherwise remain underutilized. Complementarily, thermochemical pathways—such as pyrolysis and gasification—offer viable routes for converting more recalcitrant fractions into bioenergy and platform chemicals, therewith reinforcing cascading use strategies within circular systems [112,113,114].

The pragmatic implementation of these valorization pathways increasingly depends on digital technologies embedded at the process level. High-tech sensors, IoT infrastructure, and data-driven models can support immediate monitoring of fermentation dynamics, optimize extraction yields, and anticipate maintenance needs in bioprocessing units. By improving process reliability and operational reliability, these tools improve economic performance and asset efficiency. In parallel, the recovery of nutrients through anaerobic digestion and wastewater treatment systems enables nitrogen, phosphorus, and organic matter to be reintroduced into agricultural cycles, strengthening the regenerative dimension of waste valorization strategies [26,115].

Although these biorefinery-based routes show notable promise, many remain at intermediate Technology Readiness Levels (TRLs). Challenges related to feedstock heterogeneity, process scale-up, and general economic viability continue to limit broader industrial deployment. For this reason, the combined application of techno-economic analysis and life cycle assessment remains necessary for assessing trade-offs and demonstrating the sustained viability of multi-product biorefineries, particularly those aiming to simultaneously produce nutraceuticals, biochemicals, and bioenergy from food waste streams [115,116].

### 5.2. Pillar 2—Digitization of the Food Chain

The digitalization of the food chain has played a strategic role in enabling the CE by allowing real-time monitoring of resources, strengthening traceability, and creating more intelligent, more sustainable production environments [117]. The structuring elements of this pillar focus on the application of emerging technologies for process optimization, transparency enhancement, and the connection between operational efficiency and social responsibility. Grouped into two thematic axes, these elements support a digitally oriented approach to circularity. In the studies analyzed, strong convergence is observed regarding the role of digital technologies as infrastructures that facilitate circularity, particularly by improving the visibility of material flows and supporting decision-making processes.

The axis of automation and intelligent resource monitoring encompasses practices that use advanced technologies to measure, control, and optimize the consumption of critical inputs throughout the production chain (#E7, #E9). These solutions include sensors, integrated systems, and automation resources that enable the real-time tracking of environmental performance indicators, promoting continuous adjustments and efficiency gains. Furthermore, the use of Industry 4.0 technologies contributes to more accurate management of byproducts and waste, enabling leaner, more connected production flows. This axis also integrates the adoption of business models that combine technological innovation with Corporate Social Responsibility practices, focusing on material reuse and the promotion of workers’ and community well-being [94]. These elements recur across many studies, indicating their empirical relevance and reinforcing their role as important facilitators of circular practices in digitally supported production systems.

The axis of traceability and digital transparency refers to the digitalization of production and logistics processes, incorporating technologies such as blockchain, IoT, and augmented reality to ensure the traceability of food products (#E12). These tools enable the accurate recording of product origins, logistics routes, and environmental impacts, facilitating consumer access to reliable information and strengthening trust across the value chain. In addition to enhancing food safety, digitalization at this level allows companies to clearly communicate their environmental commitments and increase accountability for the life cycles of their products [53,105].

On the bioindustrial side, digitalization of the food chain is a strategic means to support CE practice and contributes to the operational complexities associated with biorefinery-based systems. All integrated biorefineries depend on seamless coordination among heterogeneous biomass streams, diverse conversion routes, and variable process conditions. Digital technologies here function as enablers or infrastructures that enhance the visibility of material flows, energy consumption, and process performance in real time, thus enabling circular practices to be developed and operated much more accurately and reliably [118,119].

In biorefinery settings, automation and intelligent resource monitoring become vital to the sustainability of fermentation, enzymatic conversion, separation, and energy recovery activities in highly regulated environments. The sensors, integrated control systems, and data-driven monitoring tools could help fine-tune temperature, pH, residence time, and substrate utilization, minimize losses, and optimize biomass valorization routes. Through greater control over by-product and waste streams, digitalization reinforces the link between food processing and downstream biorefinery by embedding leaner, more connected circular production flows [67,120].

Similarly, digital traceability and transparency tools are gaining importance beyond consumer applications, enabling governance and accountability across bio-based value chains. Digitalized systems of traceability can track the source, processing route, and environmental performance of biomass in a biorefinery network of suppliers and support coordination among food producers, biorefineries, and other partners [121,122].

### 5.3. Pillar 3—Sustainable Education and Stakeholder Engagement

The transition toward circular systems in the food industry requires technological and operational innovations accompanied by cultural and behavioural changes. The engagement of consumers, employees, and other actors in the chain emerges from effective communication strategies, educational programs, and transparent channels for dialogue. This pillar organizes the structuring elements into two complementary thematic axes: education for circularity and informational engagement. It is possible to verify, in the delimited studies, that behavioural change and stakeholder awareness act as fundamental bridges for the transition of the EC, since technology, by itself, does not affect consumer perception and organizational culture, as consumers’ pro-sustainability attitudes do not necessarily translate into actual purchasing behaviour, reflecting a persistent intention–behaviour gap in sustainable consumption [54,123,124]. However, there are significant variations in the depth and scope of engagement strategies, ranging from informative approaches to more participatory and transformative initiatives.

The axis of education and awareness for responsible consumption encompasses actions that educate consumers about the impacts of waste, the benefits of circular products, and the importance of sufficiency as a guiding principle in decision-making (#E3, #E5, #E15). Strategies such as educational campaigns, environmental labelling, and the inclusion of sustainability messages on packaging have been used to promote food choices aligned with circularity. These practices are particularly effective when linked to health and well-being attributes, as demonstrated by studies on the acceptance of functional foods produced from by-products [105,123]. These elements appear in many studies, standing out as drivers of consumer alignment with circular practices, particularly when associated with perceived benefits for health and the environment [57].

The axis of technological communication and digital engagement involves providing accessible information about production processes, highlighting the use of by-products and the environmental impacts associated with food (#E21, #E30). Digital technologies such as social media, QR codes, and interactive platforms have proven to be practical tools for bringing consumers closer to sustainable organizational practices, fostering trust and active engagement. In addition to countering greenwashing, this axis encourages dialogue with society and strengthens the institutional positioning of companies committed to circularity [49,53]. While educational approaches focus on behavioural change, digital engagement strategies tend to emphasize transparency, interaction, and accessibility to information, thereby complementing trust-building and stakeholder involvement rather than directly influencing consumption patterns [49].

Educational and stakeholder engagement are essential for the social and institutional acceptance of bioindustrial circular solutions. For integrated biorefineries, unconventional feedstocks, such as food waste, by-products, or wastewater-derived biomass, are often used, which may be met with skepticism from consumers and other actors if they are not well communicated. Education projects that inform consumers about biorefinery processes, product safety, and environmental benefits also reduce perceived risk and increase the acceptance of bio-based products. Transparent communication of the origin, processing routes, and end uses of biomass streams also promotes coordination among food producers, biorefineries, regulators, and local communities. By combining knowledge sharing within a participatory context, this pillar reinforces the socio-technical foundations needed to scale biorefinery-driven CE models in the food sector [125,126,127].

### 5.4. Pillar 4—Strategic Partnerships for Circular Businesses

The transition toward circular production models in the food industry requires coordination among multiple actors. Strategic partnerships enable the sharing of technical knowledge, the joint development of sustainable solutions, and the territorial integration of circular practices. This pillar consists of structuring elements that guide the formation of collaborative arrangements among companies, research centers, and local suppliers, organized into two principal axes. In the studies analyzed, the role of interorganizational collaboration as a structural condition for circularity is evident, especially in complex, resource-intensive sectors such as the food industry. From this perspective, circular transitions do not depend solely on internal capabilities; coordinated actions among supply chain actors are also necessary [20,128,129].

The axis of technological and institutional partnerships encompasses collaborations with biorefineries, universities, R&D centers, and other organizations within the innovation ecosystem focused on by-product reuse and energy conversion from waste (#E11, #E18). The initiatives described range from anaerobic digestion in local biorefineries for renewable energy production to the formation of interorganizational collaboration networks to finance, test, and scale circular solutions. Such arrangements strengthen the technical capacity and resilience of organizations while generating positive externalities for both the territory and the institutional environment in which they operate [64,93].

The axis of territorial reconfiguration of the supply chain focuses on building shorter, more resilient, and sustainable supply chains by prioritizing local suppliers and reterritorializing production (#E23, #E27). The proximity-based logic seeks to minimize logistical emissions, reduce transportation costs, and foster local economies. Moreover, it reinforces the synergy between environmental sustainability and regional development, contributing to more autonomous and less vulnerable food systems [64,130].

From a bioindustrial perspective, strategic partnerships play a decisive role in enabling the deployment of integrated and multi-product biorefineries, which typically go beyond the technical, financial, and organizational capacity of individual firms. The implementation of these systems depends on coordinated investments, shared access to biomass streams, and effective alignment among food producers, biorefineries, technology providers, and public institutions. In this context, territorially based partnerships facilitate the aggregation of heterogeneous feedstocks, improve logistical efficiency, and support the alignment of regulatory and sustainability objectives [127,131,132].

### 5.5. Pillar 5—Regenerative Practices and Renewable Resources

The incorporation of regenerative practices and the strategic use of renewable resources are essential to reversing environmental impacts and building food systems that are more resilient, biodiverse, and self-sustaining [133]. This pillar is structured around structuring elements aimed at regenerating productive ecosystems, reducing chemical inputs, and converting waste into energy and biomass, organized into two interdependent axes. The analyzed studies that comprised this pillar address the growing importance of regenerative approaches to improve circular systems, especially in light of the environmental limitations identified in efficiency-based models. In this sense, it is necessary to go beyond waste reduction, seeking the restoration of ecological functions and the regeneration of resources in the long term [64,93].

The axis of ecological recovery and systemic efficiency brings together agricultural practices that promote soil health, the sustainable use of natural resources, and the redesign of production in line with degrowth principles (#E10, #E13, #E26). These actions include replacing chemical inputs with agroecological strategies, fostering functional biodiversity, and using statistical models to address uncertainties in demand and resource allocation. In addition to increasing the efficiency of material flows, these approaches also challenge the paradigm of unlimited growth and guide organizations toward achieving a balance between environmental performance and value creation over time [53,64].

The axis of bioenergy and regenerative waste valorization focuses on initiatives that transform waste into renewable energy, biomass, and productive inputs through advanced technologies (#E4, #E14, #E17, #E28). Among these initiatives, automated composting, pyrolysis for biofuel production, and the use of waste-fed microalgae to generate lipids, carbohydrates, and functional biomass stand out. Algae cultivation is also highlighted as a strategy for carbon sequestration and for reducing pressure on arable land. These practices, in addition to being regenerative, enhance circularity by linking energy solutions with routes for by-product reuse [93,134].

From the perspective of bioindustrial, regenerative activities and the exploitation of renewable resources are closely associated with the development of integrated biorefinery systems, which strive to close the loops of materials, energy, and nutrients. Emerging technologies like anaerobic digestion, thermochemical conversion, and algae-based platforms enable the recovery of energy and nutrients from organic residues, supporting agricultural regeneration and industrial sustainability. Integration of bioenergy and biomass valorization along these routes into circular food systems can reduce dependence on fossil fuels, improve overall system resilience, and foster synergies among food production, energy generation, and ecosystem restoration [67,127].

## 6. Conclusions

This study aimed to propose strategic pillars to guide the adoption of the CE in the food industry, based on the systematization of structuring elements identified in the scientific literature. Through a content analysis, thirty structuring elements of circularity were identified and organized into five pillars: valorization of waste and by-products, digitalization of the food chain, sustainable education and stakeholder engagement, strategic partnerships for circular businesses, and regenerative practices and renewable resources.

In doing so, the study directly addresses the research gap in the fragmentation between strategic CE approaches and their operational implementation, particularly in biorefinery-based systems. The proposed pillars provide a structured conceptual framework that articulates circular practices within the specific context of the food industry, thereby offering a coherent pathway for transitioning from dispersed initiatives to integrated circular strategies.

In this way, the research question of how to structure strategic CE actions in the food industry was answered using evidence from the literature. The developed pillars also help fill the scientific gap by providing applicable frameworks that articulate circular practices within the specific context of the food sector.

The main scientific contribution of this study is the organization of a dispersed body of information on CE in the food industry. By integrating documented practices and proposing a functional categorization, this work deepens the field’s theoretical foundation and supports further investigations into the application of circularity in food supply chains. From an applied perspective, the study offers an instructional tool for food industry companies seeking to align their operations with the principles of the CE. The proposed pillars provide practical guidelines that managers and professionals in the sector can operationalize to reduce waste, valorize residues, foster territorial partnerships, and incorporate regenerative technologies. In doing so, it contributes to the materialization of commitments aligned with the Sustainable Development Goals (SDGs), particularly SDGs 2, 7, 9, 12, 13, and 17. In this sense, it is important to emphasize that the sustainability value of this study does not directly measure environmental or social performance indicators, as it is limited to supporting the implementation of CE practices. By structuring and systematizing key elements, the study enables organizations to improve resource efficiency, reduce waste generation, and strengthen circular value chains, thereby achieving more effective sustainability outcomes in practice. This positions the study as an enabler of sustainability, rather than a direct measurement of sustainability performance.

Furthermore, this study illustrates the role of biorefinery systems as essential operational enablers for implementing CE principles in the food industry. Biorefineries provide the infrastructure required to convert diverse waste streams into high-value products, including functional ingredients, bio-based chemicals, and bioenergy, thereby linking food processing with biomass conversion pathways. The effectiveness of the proposed pillars is therefore strengthened when situated within integrated, multi-product biorefinery systems able to support circularity at scale.

For future studies, it is recommended to empirically validate the framework across different organizational contexts through case studies and sectoral analyses. In particular, future research may examine the magnitude of CE practices’ contributions to specific sustainability outcomes, as well as the contextual conditions and potential trade-offs associated with their implementation. Expanding the documentary base to include sustainability reports, technical standards, and institutional documents can strengthen the robustness of the proposed analytical structure. It is also suggested that the feasibility of applying the proposed pillars to small and medium-sized enterprises be investigated to enhance the proposal’s scalability and applicability across diverse productive realities.

## Figures and Tables

**Table 1:** Comparative techno-environmental assessment of biorefinery pathways.

Conversion Pathway	Main Outputs	Energy Intensity	GHG Emissions	Economic Feasibility	Key Operational Challenge
Fermentation	Bioethanol, organic acids	Medium	Low	Moderate–High	Feedstock variability
Anaerobic digestion	Biogas, digestate	Low	Very low	High	Limited product diversity
Pyrolysis/gasification	Bio-oil, syngas, biochar	High	Medium	Moderate	High energy demand
Extraction (bioactives)	Nutraceuticals, functional compounds	Medium	Low	High (value-added products)	High processing cost and purification complexity

Source: Compiled by the authors based on literature synthesis, including [29,69,70,72,73,74,75,76].

**Table 2:** Structuring Elements of the Circular Economy in the Food Industry.

#	Structuring Elements	Axes	Frequency
1	Integrate CE practices into the food supply chain to reduce waste at all stages, from agricultural production to final consumption, and to create collection and resource recovery systems to reuse food waste in new production cycles.	SDG 12	29
2	Promote the use of agricultural by-products and waste to generate value throughout the production chain, whether through animal feed, optimized biomass utilization, or bioenergy generation, thereby contributing to a more efficient and sustainable system.	SDG 2	23
3	Encourage education and awareness among consumers and supply chain members about the benefits and importance of CE, thereby raising stakeholder awareness about waste reduction programs and recycling implementation.	SDG 3	10
4	Invest in technologies that enable advanced recycling of agricultural waste, such as automated composting to produce natural fertilizers and pyrolysis to convert biomass into biofuels.	SDG 7	10
5	Promote business models that encourage responsible consumption and waste reduction, including the use of sustainable packaging that extends food shelf life.	SDG 12	10
6	Promote the use of biodegradable or recyclable packaging materials to minimize environmental impact and reduce waste in the food supply chain.	SDG 12	7
7	Implement advanced technological solutions to monitor the consumption of critical resources, promoting efficiency and sustainability in the production chain. At the same time, adopt circular business models that integrate Corporate Social Responsibility practices, focusing on the reuse and recycling of materials for the well-being of workers and the community.	SDG 8	7
8	Set goals for reducing waste and increasing the use of by-products, establish the actions necessary to achieve them, and monitor progress through key performance indicators (KPIs), disclosing advances in sustainability reports.	SDG 12	7
9	Promote the use of Industry 4.0 technologies to optimize resource and by-product management in food production.	SDG 9	6
10	Invest in research and development of sustainable production models that improve resource management, using statistical approaches to address uncertainties in demand and rates of return, and promote the efficiency of material flows in a CE system.	SDG 9	6
11	Establish partnerships with local biorefineries that use anaerobic digestion to convert food waste into renewable energy, thereby maximizing energy recovery and advancing the CE.	SDG 7	6
12	Digitize production processes and the food supply chain, using technologies such as blockchain, IoT, and augmented reality to improve traceability, food safety, and sustainability, ensuring that consumers have access to accurate and reliable information about the origin and environmental impact of food products.	SDG 9	5
13	Promote agricultural practices that reduce chemical use, support biodiversity, and enhance soil resilience, thereby positively impacting the health and well-being of workers and consumers.	SDG 2	5
14	Promote the cultivation of algae in aquatic environments to reduce the need for arable land and help mitigate global warming through carbon sequestration. Invest in technologies that use wastewater to cultivate algae, transforming it into high-quality biomass for the production of nutritious foods and bioenergy.	SDG 13	4
15	Develop educational campaigns that explain the environmental and health benefits of reusing food byproducts in the production of new functional foods, increasing consumer acceptance of these new products.	SDG 3	4
16	Promote packaging made from biodegradable and compostable materials, such as bioplastics and polylactic acid (PLA), to minimize environmental impact and meet consumer preferences, thereby reducing plastic waste.	SDG 12	4
17	Develop bioreactor technologies that use waste to feed microalgae, enabling the efficient production of food and biomass. Microalgae fed on this waste produce lipids and carbohydrates that can be used in food manufacturing and bioenergy generation.	SDG 7	4
18	To establish partnerships with other companies in the food sector, universities, and R&D centers to share knowledge, technologies, and best practices for the use of by-products, and to consider public–private partnerships for financing and technical support.	SDG 17	3
19	Using insects to process food and agricultural waste, transforming it into protein sources for animal feed and potentially for human consumption, contributes to a more sustainable, circular food production system.	SDG 2	3
20	Invest in technologies that use green solvents to obtain natural pigments from food waste, replacing synthetic dyes.	SDG 12	3
21	Provide information on how innovative food technologies are used to create food from by-products, emphasizing sustainability and health aspects.	SDG 3	2
22	Establish internal programs dedicated to identifying and utilizing byproducts from primary production, including creating teams or committees focused on R&D for new products from waste, such as fruit and vegetable peels.	SDG 12	2
23	Establish partnerships with local suppliers to minimize emissions associated with product transportation, thereby reducing CO2 emissions and supporting local economies, prioritizing closer supply chains to reduce logistics costs.	SDG 13	2
24	Adopt analytical tools for the organization’s departments to improve transparency and efficiency in the management of waste and byproducts, enabling food-sector companies to identify and reduce hidden costs associated with material and energy waste.	SDG 9	2
25	Establish mechanisms for the collection and valorization of non-edible by-products, such as feathers, blood, and slaughterhouse waste, for use in non-food sectors, transforming what would otherwise be waste into sources of revenue.	SDG 15	2
26	Adopting degrowth principles, redefining business success in terms of sustainability, reducing environmental impacts, limiting the use of natural resources, and focusing on the efficiency of existing processes.	SDG 12	2
27	Prioritize re-territorializing production to favor proximity to local suppliers, aiming to reduce transportation costs, lower the carbon footprint, support local economies, and increase supply chain resilience.	SDG 11	2
28	Using fruit processing by-products as raw material for biochar and bioenergy production	SDG 15	2
29	Implement industrial-scale waste-recovery processes, such as extracting fibers from fruit peels, producing proteins from legumes, and isolating antioxidants from by-products, to create functional food ingredients.	SDG 9	2
30	Use social media as key tools to promote dialogue and engagement around the CE in the agri-food sector	SDG 12	2

## Data Availability

No new data were created or analyzed in this study. Data sharing is not applicable to this article.

## Appendix A

**Table A1:** The 30 Most Cited Articles on CE in Food Industry from 2019 to 2024.

#	Title	Author	Journal	Citation
1	Barriers to circular food supply chains in China	[20]	Supply Chain Management	184
2	Sustainable consumption in the circular economy. An analysis of consumersâ€™ purchase intentions for waste-to-value food	[123]	Journal of Cleaner Production	182
3	The role of farm animals in a circular food system	[91]	Global Food Security	162
4	Algae and their potential for a future bioeconomy, landless food production, and the socio-economic impact of an algae industry	[93]	Organic Agriculture	67
5	Innovative processes in managing an enterprise from the energy and food sector in the era of industry 4.0	[94]	Processes	63
6	Circular economy and corporate social responsibility in the agricultural system: Cases study of the Italian agri-food industry	[128]	Agricultural Economics (Czech Republic)	59
7	Consumersâ€™ attitude towards food by-products: the influence of food technology neophobia, education and information	[57]	International Journal of Food Science and Technology	55
8	Nested circularity in food systems: A Nordic case study on connecting biomass, nutrient and energy flows from field scale to continent	[135]	Resources, Conservation and Recycling	56
9	A fuzzy multi-objective optimization model for sustainable closed-loop supply chain network design in food industries	[136]	Environment, Development and Sustainability	50
10	Suffciency business strategies in the food industry-the case of oatly	[137]	Sustainability (Switzerland)	47
11	Analysis of Waste Minimization Challenges to European Food Production Enterprises	[95]	Emerging Science Journal	35
12	From orange juice by-product in the food industry to a functional ingredient: Application in the circular economy	[51]	Foods	35
13	Coming out the egg: Assessing the benefits of circular economy strategies in agri-food industry	[52]	Journal of Cleaner Production	30
14	Plastics and sustainable purchase decisions in a circular economy: The case of Dutch food industry	[105]	PLoS ONE	24
15	Combining land-based organic and landless food production: a concept for a circular and sustainable food chain for Africa in 2100	[92]	Organic Agriculture	24
16	The socio-economic force field of the creation of short food supply chains in Europe	[138]	Journal of Food and Nutrition Research	23
17	Material flow cost accounting (MFCA) to enhance environmental entrepreneurship in the meat sector: Challenges and opportunities	[21]	Journal of Environmental Management	22
18	The future role of reverse logistics as a tool for sustainability in food supply chains: a Delphi-based scenario study	[22]	Supply Chain Management	20
19	Assessment of the sustainability of the European agri-food sector in the context of the circular economy	[48]	Sustainable Production and Consumption	19
20	Consumer perception of the circular economy concept applied to the food domain: An exploratory approach	[54]	Sustainability (Switzerland)	18
21	Dry Anaerobic Digestion of Food Industry by-Products and Bioenergy Recovery: A Perspective to Promote the Circular Economy Transition	[102]	Waste and Biomass Valorization	16
22	Introducing a degrowth approach to the circular economy policies of food production, and food loss and waste management: Towards a circular bioeconomy	[64]	Sustainability (Switzerland)	15
23	Filamentous fungi for sustainable vegan food production systems within a circular economy: Present status and future prospects	[107]	Food Research International	14
24	Grape pomace as an energy source for the food industry: A thermochemical and kinetic analysis	[47]	Food and Bioproducts Processing	13
25	Actions needed before insects can contribute to a real closed-loop circular economy in the EU	[23]	Journal of Insects as Food and Feed	10
26	A feasibility study on green biorefinery of high lignin content agro-food industry waste through supercritical water treatment	[103]	Journal of Cleaner Production	10
27	Circular Economy for Food Industry Waste: Development and Characterization of Spray-Dried Acid Whey Encapsulated in Millet Matrix	[130]	ACS Food Science and Technology	9
28	Greener technologies in agri-food wastes valorization for plant pigments: Step towards circular economy	[104]	Current Research in Green and Sustainable Chemistry	9
29	Social media on the route to circular economy transition from a dialogic perspective: evidence from the agri-food industry	[49]	British Food Journal	8
30	In the nexus of sustainability, circular economy and food industry: Circular food package design	[53]	Journal of Cleaner Production	8

**Table A2:** Structuring Elements of the Circular Economy in the Food Industry.

#	Structuring Elements/Articles	1 [20]	2 [123]	3 [91]	4 [93]	5 [94]	6 [128]	7 [57]	8 [135]	9 [136]	10 [137]	11 [95]	12 [51]	13 [52]	14 [105]	15 [92]	16 [138]	17 [21]	18 [22]	19 [48]	20 [54]	21 [102]	22 [64]	23 [107]	24 [47]	25 [23]	26 [103]	27 [130]	28 [104]	29 [49]	30 [53]
1	Integrate CE practices into the food supply chain to reduce waste at all stages, from agricultural production to final consumption, and to create collection and resource recovery systems to reuse food waste in new production cycles.	x	x	x	x		x	x	x	x	x	x	x	x	x	x	x	x	x	x	x	x	x	x	x	x	x	x	x	x	x
2	Promote the use of agricultural by-products and waste to generate value throughout the production chain, whether through animal feed, optimized biomass utilization, or bioenergy generation, thereby contributing to a more efficient and sustainable system.		x	x	x		x	x	x	x		x	x	x	x	x		x	x	x	x	x		x	x	x	x	x		x	
3	Encourage education and awareness among consumers and supply chain members about the benefits and importance of CE, thereby raising stakeholder awareness about waste reduction programs and recycling implementation.	x						x			x	x	x		x		x	x			x									x	
4	Invest in technologies that enable advanced recycling of agricultural waste, such as automated composting to produce natural fertilizers and pyrolysis to convert biomass into biofuels.				x		x		x	x				x		x						x			x		x		x		
5	Promote business models that encourage responsible consumption and waste reduction, including the use of sustainable packaging that extends food shelf life.		x				x	x			x	x			x					x	x		x								x
6	Promote the use of biodegradable or recyclable packaging materials to minimize environmental impact and reduce waste in the food supply chain.	x								x	x			x	x													x			x
7	Implement advanced technological solutions to monitor the consumption of critical resources, promoting efficiency and sustainability in the production chain. At the same time, adopt circular business models that integrate Corporate Social Responsibility practices, focusing on the reuse and recycling of materials for the well-being of workers and the community.					x	x			x	x	x						x		x											
8	Set goals for reducing waste and increasing the use of by-products, establish the actions necessary to achieve them, and monitor progress through key performance indicators (KPIs), disclosing advances in sustainability reports.						x			x		x	x					x	x	x											
9	Promote the use of Industry 4.0 technologies to optimize resource and by-product management in food production.	x		x	x	x												x	x												
10	Invest in research and development of sustainable production models that improve resource management, using statistical approaches to address uncertainties in demand and rates of return, and promote the efficiency of material flows in a CE system.					x				x	x									x							x		x		
11	Establish partnerships with local biorefineries that use anaerobic digestion to convert food waste into renewable energy, thereby maximizing energy recovery and advancing the CE.				x				x					x		x			x			x									
12	Digitize production processes and the food supply chain, using technologies such as blockchain, IoT, and augmented reality to improve traceability, food safety, and sustainability, ensuring that consumers have access to accurate and reliable information about the origin and environmental impact of food products.	x	x	x		x											x														
13	Promote agricultural practices that reduce chemical use, support biodiversity, and enhance soil resilience, thereby positively impacting the health and well-being of workers and consumers.			x			x				x								x	x											
14	Promote the cultivation of algae in aquatic environments to reduce the need for arable land and help mitigate global warming through carbon sequestration. Invest in technologies that use wastewater to cultivate algae, transforming it into high-quality biomass for the production of nutritious foods and bioenergy.				x				x							x							x								
15	Develop educational campaigns that explain the environmental and health benefits of reusing food byproducts in the production of new functional foods, increasing consumer acceptance of these new products.		x					x					x								x										
16	Promote packaging made from biodegradable and compostable materials, such as bioplastics and polylactic acid (PLA), to minimize environmental impact and meet consumer preferences, thereby reducing plastic waste.													x	x				x												x
17	Develop bioreactor technologies that use waste to feed microalgae, enabling the efficient production of food and biomass. Microalgae fed on this waste produce lipids and carbohydrates that can be used in food manufacturing and bioenergy generation.				x				x							x						x									
18	To establish partnerships with other companies in the food sector, universities, and R&D centers to share knowledge, technologies, and best practices for the use of by-products, and to consider public–private partnerships for financing and technical support.			x			x						x																		
19	Using insects to process food and agricultural waste, transforming it into sources of protein for animal feed and potentially for human consumption, contributes to a more sustainable and circular food production system.															x						x				x					
20	Invest in technologies that use green solvents to obtain natural pigments from food waste, replacing synthetic dyes.										x				x														x		
21	Provide information on how innovative food technologies are used to create food from by-products, emphasizing sustainability and health aspects.							x															x								
22	Establish internal programs dedicated to identifying and utilizing byproducts from primary production, including creating teams or committees focused on R&D for new products from waste, such as fruit and vegetable peels.												x						x												
23	Establish partnerships with local suppliers to minimize emissions associated with product transportation, thereby reducing CO2 emissions and supporting local economies, prioritizing closer supply chains to reduce logistics costs.													x			x														
24	Adopt analytical tools for the organization’s departments to improve transparency and efficiency in the management of waste and byproducts, enabling food-sector companies to identify and reduce hidden costs associated with material and energy waste.																	x	x												
25	Establish mechanisms for the collection and valorization of non-edible by-products, such as feathers, blood, and slaughterhouse waste, for use in non-food sectors, transforming what would otherwise be waste into sources of revenue.													x				x													
26	Adopting degrowth principles, redefining business success in terms of sustainability, reducing environmental impacts, limiting the use of natural resources, and focusing on the efficiency of existing processes.										x												x								
27	Prioritize re-territorializing production to favor proximity to local suppliers, aiming to reduce transportation costs, lower the carbon footprint, support local economies, and increase supply chain resilience.																x						x								
28	Using fruit processing by-products as raw material for biochar and bioenergy production												x												x						
29	Implement industrial-scale waste-recovery processes, such as extracting fibers from fruit peels, producing proteins from legumes, and isolating antioxidants from by-products, to create functional food ingredients.												x															x			
30	Use social media as key tools to promote dialogue and engagement around the CE in the agri-food sector														x															x	

Note: “x” indicates that the corresponding article addresses the respective structuring element identified through the content analysis.
